# Assessing the spreading potential of an undetected case of COVID-19 in orthopaedic surgery

**DOI:** 10.1007/s00402-020-03516-1

**Published:** 2020-06-10

**Authors:** K. N. Schneider, C. L. Correa-Martínez, G. Gosheger, C. Rickert, D. Schorn, A. Mellmann, V. Schwierzeck, S. Kampmeier

**Affiliations:** 1 Department of Orthopaedics and Tumor Orthopaedics, University Hospital of Münster, Albert-Schweitzer-Campus 1, 48149 Münster, Germany; 2 Institute of Hygiene, University Hospital of Münster, Albert-Schweitzer-Campus 1, 48149 Münster, Germany

**Keywords:** COVID-19, SARS-CoV-2, MDRO, Nosocomial, Infection, Orthopaedics

## Abstract

**Background:**

With the novel coronavirus-induced disease (COVID-19), there is the fear of nosocomial infections and severe acute respiratory syndrome coronavirus 2 (SARS-CoV-2) transmissions to healthcare workers (HCW). We report the case of a 64-year-old male patient who underwent explantation of a shoulder prosthesis due to a periprosthetic infection. He was tested SARS-CoV-2 positive 7 days after admission to the orthopaedic department following strict infection control measures, routinely including screening all patients for multi-drug-resistant organism (MDRO) colonization upon admission. Aim of our study is to report on the spreading potential of SARS-CoV-2 in a healthcare setting if standard contact precautions and infection control measures have been established.

**Methods:**

All HCW with exposure to the patient from day of admission until confirmed diagnosis of COVID-19 were identified and underwent oropharyngeal swab testing for SARS‐CoV‐2 by real-time RT-PCR.

**Results:**

Sixty-six HCW were identified: nine orthopaedic surgeons, four anaesthesiologists, 25 orthopaedic nurses, five nurse anesthetists, eight scrub nurses, five nursing students, two medical assistants and seven service employees. Fourteen HCW (21%) showed clinical symptoms compatible with a SARS-CoV-2 infection: cough (*n* = 4), sore throat (*n* = 3), nasal congestion (*n* = 3), dyspnea (*n* = 2), fever (*n* = 1), headache and myalgia (*n* = 1). SARS-CoV-2 was not detected in any of the 66 HCW.

**Conclusion:**

Hygienic measures and contact precautions, aimed at preventing the spread of MRDO, may have helped to prevent a SARS-CoV-2 transmission to HCW—despite high-risk exposure during intubation, surgical treatment and general care.

**Level of evidence:**

IV, case series.

## Background

In March 2020, the World Health Organization (WHO) has declared the outbreak of the novel coronavirus-induced disease (COVID-19) caused by the severe acute respiratory syndrome coronavirus 2 (SARS-CoV-2) a global pandemic [1]. As of April 21, 2020, there are 2,314,621 confirmed cases of COVID-19 in 213 countries [2]. While estimating case fatality rates for COVID-19 is challenging, there is a consensus that substantially higher rates are reported amongst older age groups [3, 4]. The basic reproduction number (*R*_0_) represents the average number of new infections generated by one infected person and is expected to be between two and three in COVID-19 [5], but a suspected asymptomatic ratio of 30% complicates early identification and isolation of potential SARS-CoV-2 spreaders [6]. Especially, SARS-CoV-2 transmission to healthcare workers (HCW) is feared as it embodies an enormous spread potential to patients and co-workers [7, 8].

While hygienic measures to prevent nosocomial SARS-CoV-2 transmission are gradually implemented, infection control measures to limit the spread of multi-drug-resistant organisms (MDRO) have long been enforced and routinely applied in varying extents.

We report the case of a 64-year-old male patient who was referred to our orthopaedic department with a periprosthetic shoulder infection, kept under routine preemptive MDRO isolation and tested SARS-CoV-2 positive 7 days after admission.

Aim of our study is to investigate the spread potential to HCW of SARS-CoV-2 originating from a previously undetected case, where contact precautions and infection control measures for MDRO prevention were established.

## Methods

### Infection-control measures

Over the past weeks, various infection-control measures have been enforced to prevent the spread of SARS-CoV-2: for Germany, the Federal Ministry of Health declared a temporary ban on elective surgery and outpatient clinics on March 12 [9]. Furthermore, our university hospital enforced rigorous visitor restrictions on March 20 and made wearing facemasks compulsory for all HCW on March 23. On April 02, all patients who were discharged to a rehabilitation clinic or retirement home had to undergo previous SARS-CoV-2 oropharyngeal swab testing and as of April 14, all in- and out-patients with COVID-19-related symptoms had to undergo SARS-CoV-2 swab testing prior admission with daily re-evaluation of symptoms. However, due to limited test capacities, there are neither compulsory tests for asymptomatic patients prior to admission nor routine tests among asymptomatic HCW without known exposure to a SARS-CoV-2-positive patient in our university hospital as of April 16, 2020.

**Fig. 1:**
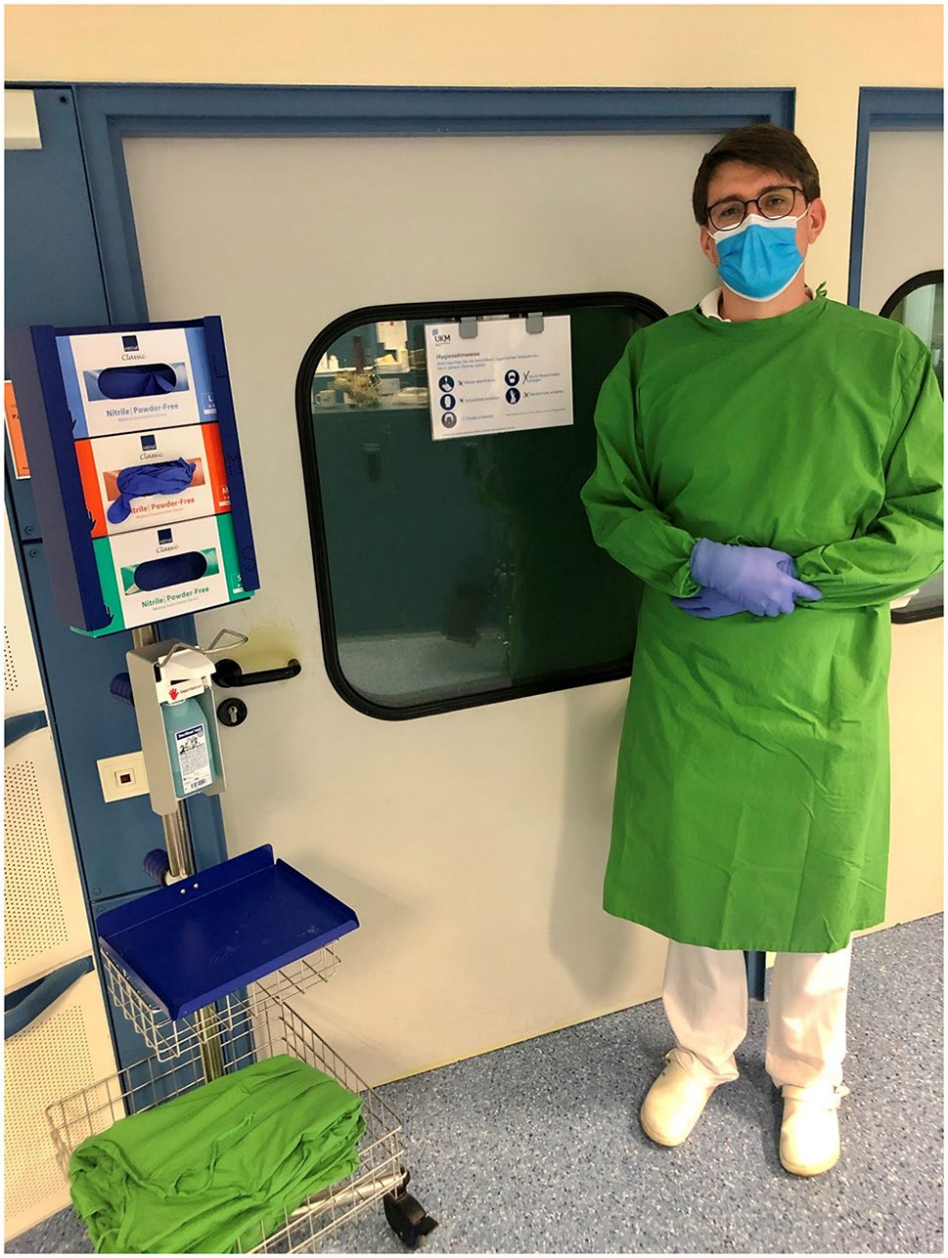
Hygienic measures to prevent the spread of MDRO include protective isolation in a single bedroom as well as compulsory facemask, gloves and protective gown for HCW

Infection-control measures to manage and prevent the spread of MDRO have routinely been implemented as standard care in our high volume surgical facility. In our department, this includes obligatory nasopharyngeal swab testing for methicillin-resistant *Staphylococcus aureus* (MRSA) and rectal swab testing for detection of vancomycin-resistant enterococci (VRE). Patients are usually tested during final consultation prior to admission, so that test results are available before hospitalization. When test results are not available upon admission, e. g. in case of external referrals or non-elective admissions, preemptive isolation is performed until final test results are available. Contact precautions include hospitalization in a single bedroom with enclosed toilet and shower. All HCW entering the patient’s room must wear a facemask, gloves, and a protective gown (Fig. 1). During preemptive isolation, councils like radiographic examinations are kept to a minimum and the patient is advised not to leave his room for unnecessary occasions (e. g. smoking, use of shared communal facilities) but to stay indoors until final test results are available.

### Undetected case of COVID-19

A 64-year-old male patient (160 cm, 70 kg) was referred to our orthopaedic department with a periprosthetic infection of his right shoulder joint.

**Fig. 2:**
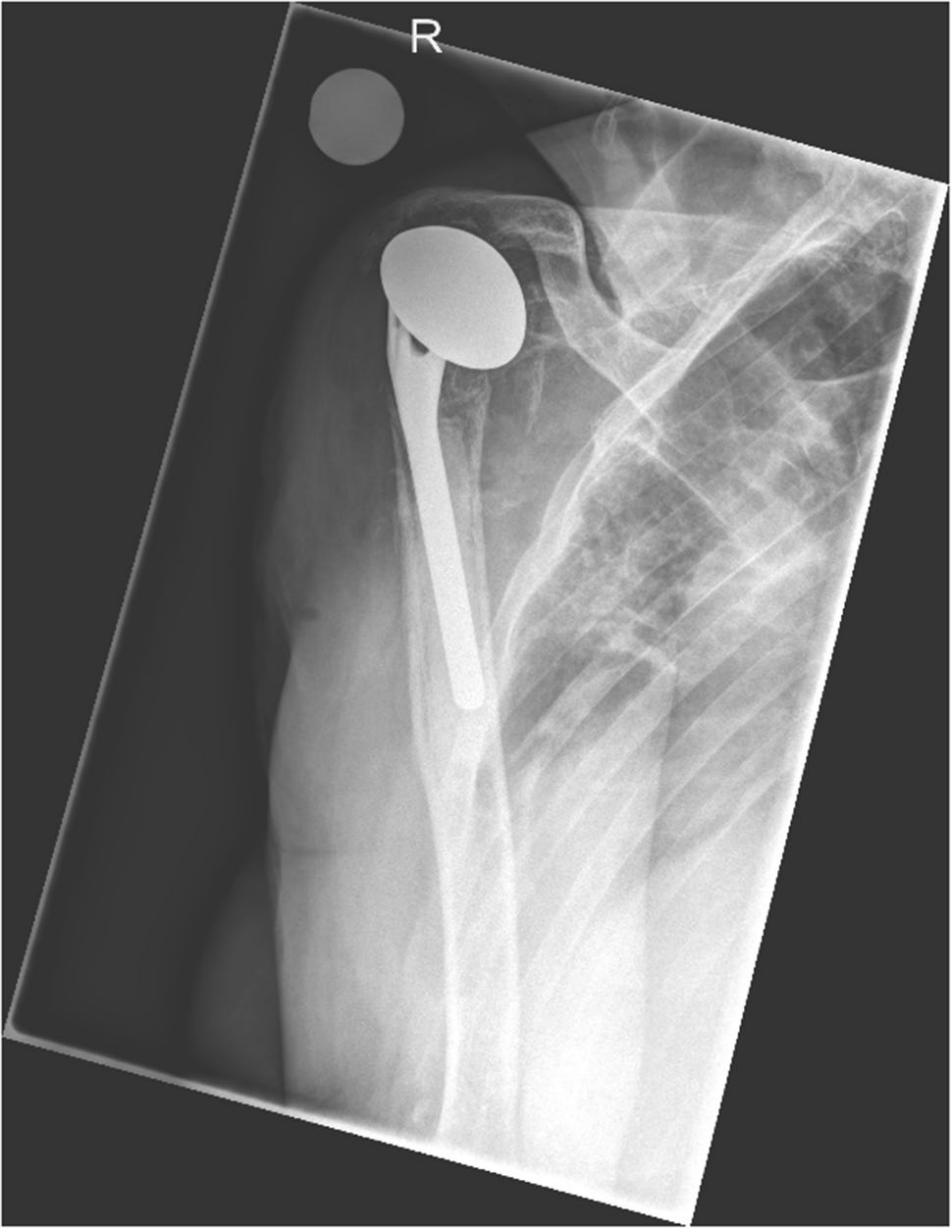
Radiographic examination of the right shoulder joint (a.p. view) prior revision surgery due to periprosthetic infection

Seven years previously, the patient suffered a dislocated four-part proximal humerus fracture following a fall and underwent subsequent open reduction and osteosynthesis with a proximal humerus internal locking system (PHILOS) plate (DePuy Synthes, West Chester, PA, USA). Four weeks postoperatively, the patient suffered another fall sustaining a comminuted proximal humeral fracture with dislocation of the PHILOS plate that was treated with a shoulder hemiprosthesis (Tornier Inc., Bloomington, MN, USA). Three weeks prior referral to our department, the patient suffered a shoulder contusion with formation of a hematoma that required surgical removal due to progressive signs of inflammation. Due to persistent signs of infection, the patient was referred to our department and clinical examination upon arrival confirmed a severe inflammation of the right shoulder joint with redness and tenderness as well as elevated inflammation parameters: C-reactive protein (CRP) of 4.2 mg/dl (normal: < 0.5 mg/dl). Radiographic examinations showed no evidence of implant failure or osteomyelitis (Fig. 2). Therefore, the diagnosis of a periprosthetic shoulder joint infection was established.

**Fig. 3:**
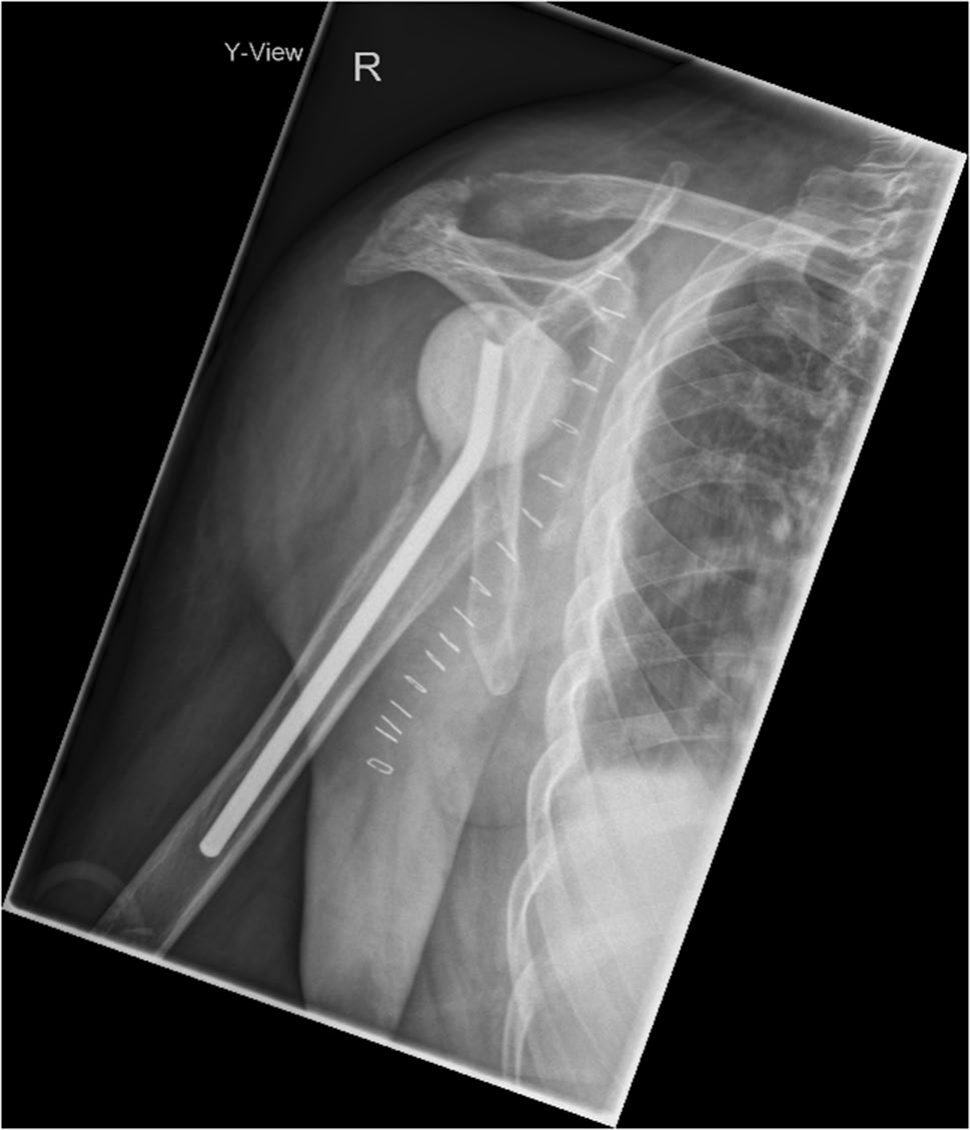
Radiographic examination of the right shoulder joint (*Y*-view), following the explantation of the hemi prosthesis and implantation of an antibiotic-impregnated bone cement spacer

Further previous medical history revealed a chronic obstructive pulmonary disease (COPD) with a history of 1020 py as well as alcohol consumption of 1–2 l white wine per day. One day after admission, the patient underwent explantation of the shoulder prosthesis, debridement and implantation of an antibiotic-impregnated bone cement spacer (Fig. 3). The patient initially received a calculated i. v. antibiotic treatment of ampicillin and sulbactam (3 g, t.i.d.) that was adjusted to cefazolin (2 g i. v., q.i.d.) after a methicillin-sensitive *Staphylococcus aureus* was detected in 5 of 5 tissue samples.

**Fig. 4:**
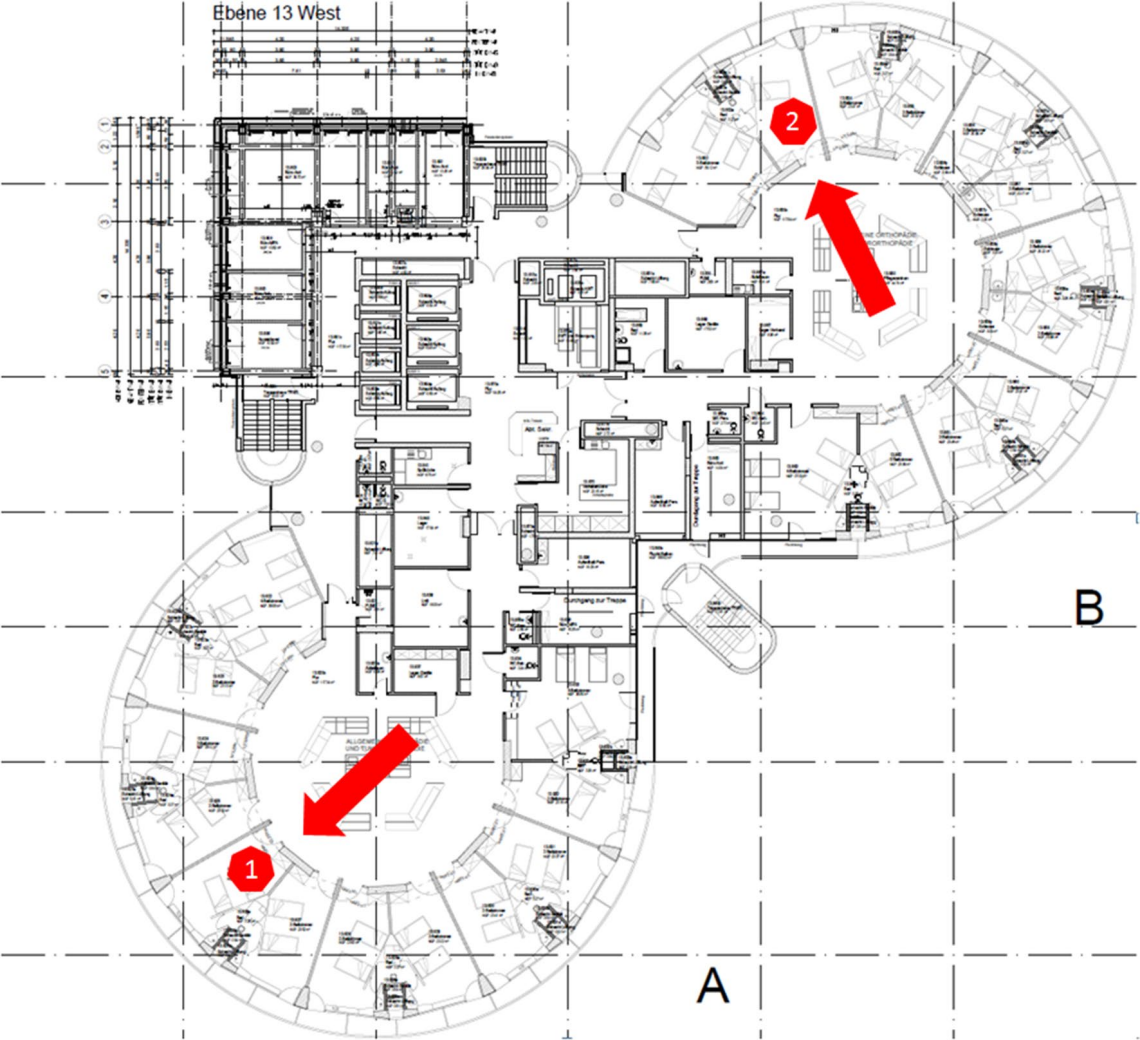
Floor plan with the single bedroom (see red arrow, 1) where the patient stayed during protective isolation and the single bedroom on the IMC unit (see red arrow, 2)

**Fig. 5:**
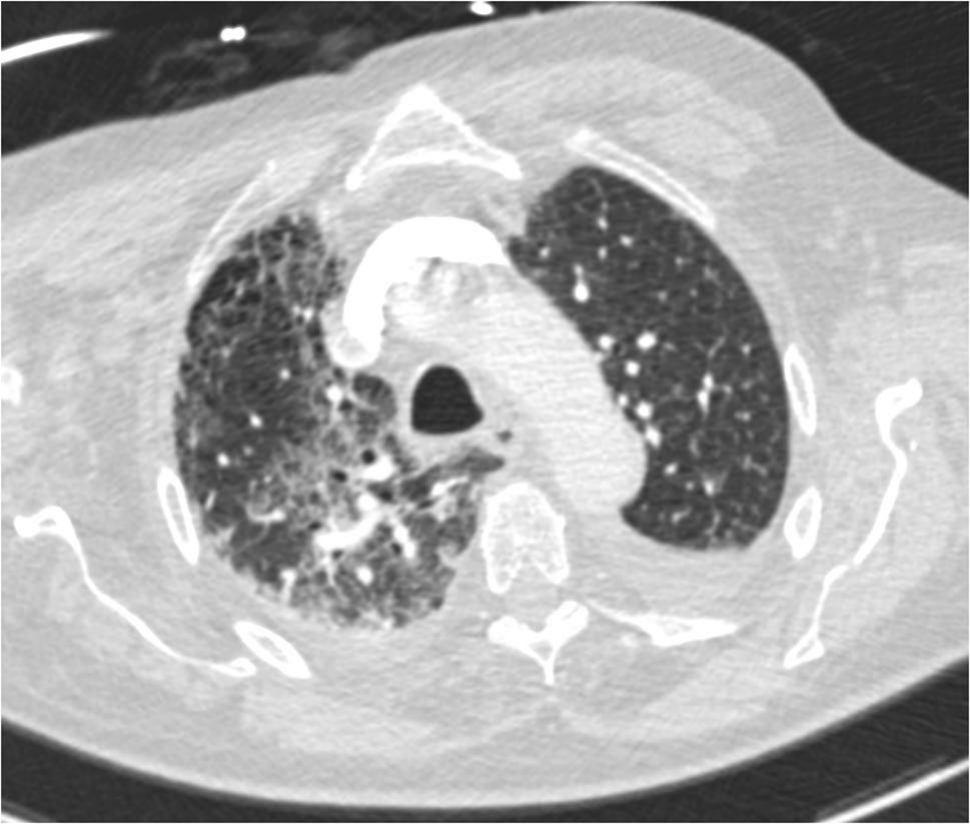
Chest computed tomography of the SARS-CoV-2-positive 64-year-old male patient with right-sided multilobe involvement and ground-glass opacity and left-sided pleural effusion

As the orthopaedic department follows strict infection control measures, the patient was placed under preventive isolation upon admission (Fig. 4, Room 1) for 4 days until negative results for MRSA and VRE colonization were available. On the same day, the negative MDRO test results arrived, the patient complained of dyspnea and was subsequently placed in a single bedroom on our intermediate care unit (IMC; Fig. 4, Room 2). Laboratory results revealed an increase in CRP to 10.4 mg/dl with auscultation and radiographic examination raising the suspicion of pneumonia. Over the following 4 days, the patient improved under symptomatic treatment and 0_2_ inhalation (4 l/min) supporting our initially suspected diagnosis of a COPD superinfection. However, respiratory distress worsened, 5 days postoperatively. An oropharyngeal swab testing was positive for SARS-CoV-2 positive, 6 days postoperatively and an additional CT-scan revealed COVID-19 typical ground-glass opacities (Fig. 5). The patient was subsequently transferred to our dedicated COVID-19 ward.

### Identifying cases

Due to the preemptive isolation of the patient, there were no potential contact patients. HCW exposed to the patient from day of admission until patient’s final diagnosis of COVID-19 were identified using work schedules and the hospital information system. All HCW were contacted, questioned for exposure as well as symptoms and categorized according to the latest identification criteria for contact persons released by the Robert Koch Institute (RKI, Table 1) [10]. Regardless of symptoms, all HCW exposed to the patient underwent oropharyngeal swab testing for SARS-CoV-2 using real-time RT-PCR [11]. HCW developing symptoms in a 14-day period after initial swab testing were scheduled for another oropharyngeal swab testing. As the patient was initially referred from an external hospital, we also followed up on potentially exposed HCW and patients in the referral hospital.

**Table 1:** Classification of contact persons according to RKI guidelines as of April 10, 2020 [10]

Category I	Category II	Category III
Cumulative face-to-face time of 15 min Direct contact to body fluids Exposed to aerosols and respiratory droplets (≤ 2 m) without use of protective gear	Cumulative face-to-face time of less than 15 min Exposed to aerosols and respiratory droplets (> 2 m) without use of protective gear	Exposed (≤ 2 m) with use of protective gear Exposed (> 2 m) without any direct contact to body fluids, aerosols and respiratory droplets

### Viral molecular testing

SARS-CoV-2 testing was performed on oropharyngeal swabs. Virus identification relied on the detection of two separate genes via real-time PCR as described previously [11]. In a first step, the envelope gene was targeted as a means of screening, followed by the RNA-dependent RNA polymerase (RdRp) gene in a second, confirmatory step [11].

## Results

Sixty-six HCW (48 female) with contact to the index patient were identified: nine orthopaedic surgeons, four anaesthesiologists, 25 orthopaedic nurses, five nurse anesthetists, eight scrub nurses, five nursing students, two medical assistants and seven service employees. Fourteen HCW (21%) reported clinical symptoms compatible with a SARS-CoV-2 infection, of which 12 HCW reported initial symptoms like cough (*n* = 4), a sore throat (*n* = 3), nasal congestion (*n* = 3), fever (*n* = 1), headache and myalgia (*n* = 1) on the day of testing. Two HCW (contact person category II) developed dyspnea (7 and 9 days after initial testing) during the 14-day period after initial swab testing. Thirteen HCW (20%) were classified as category III contact persons and 26 HCW (39%) as category II contact persons. There were 27 HCW (41%) category I contact persons: four anesthesiologists and five nurse anesthetists who performed intubation and monitoring during surgical intervention, three orthopaedic surgeons who performed surgical treatment as well as another three orthopaedic surgeons who were responsible for dressing change on the ward and 12 orthopaedic nurses who were in charge of the patient’s care on the ward. RT-PCR for SARS-CoV-2 was negative for all 66 HCW during both the initial tests and the two retests. Upon request, neither positive HCW nor patients were identified within the referral hospital.

## Discussion

With rapidly increasing cases of COVID-19 as well as regional shortages in hospital capacity and HCW, nosocomial transmission of SARS-CoV-2 may result in devastating cascading effects [7, 8, 12].

The most important findings of our study include that (1) awareness for COVID-19 should be high, even if patients are already hospitalized for several days for other conditions and had no obvious exposure to a SARS-CoV-2-positive person. (2) Despite high-risk exposure, the spreading potential of an undetected hospitalized case of COVID-19 can be kept low. (3) Contact precautions initially aimed for MDRO prevention including preemptive isolation may help to prevent intra-hospital COVID-19 infection and SARS-CoV-2 transmission.

Identification and isolation of a possible SARS-CoV-2 transmitter are challenging, because test capacities are limited and the asymptomatic ratio is reported to be as high as 30% [6, 13]. The difficulties and consequences of late diagnosis in COVID-19 have been outlined by Rong et al. who have shown that a delay in diagnosis results in a higher number of new infections (and a higher *R*_0_) [14]. They conclude that (1) early detection of cases, (2) isolation of every patient until final test result are available, and (3) contract tracing of positively tested patients are necessary to control transmission dynamics [14]. While routinely testing all admitted patients and HCW for SARS-CoV-2—regardless of symptoms—would only help in COVID-19 detection in patients, where clinical symptoms arise within the next 1–2 days, restricted test capacities currently limit screening to symptomatic persons/patients or asymptomatic persons/patients with known COVID-19 exposure. Although not tested, it is very likely that on admission this patient would have been tested negatively. The detection of the SARS-CoV-2 infection in our initially asymptomatic patient was further aggravated as postoperative respiratory symptoms with a history of COPD and 1020 py initially remained vague and were interpreted as pneumonia. Clinical improvements under treatment were misguiding and led to further delay in diagnosis which highlights that even in previously hospitalized patients without obvious exposure to a SARS-CoV-2-positive person, awareness for COVID-19 should be high and—in case of symptoms compatible with a SARS-CoV-2 infection—an infection with SARS-CoV-2 should be taken into consideration at least under the current epidemiological situation.

Transmission of SARS-CoV-2 and spreading of COVID-19 occurs during close contact with an infected patient and inhalation of respiratory droplets or aerosols during speaking, coughing or sneezing [15–17]. As there is currently no vaccine available, the best COVID-19 prevention is to avoid exposure to the virus [17]. To prevent nosocomial infections, several hygienic measures have been proposed, including sensitizing and teaching of patients and HCW, use of facemasks, regular hand washing, minimizing contacts, and maintaining a safety distance as well as protective isolation of positive-tested patients [17]. Undetected COVID-19 cases can lead to devastating intra-hospital infections: 22 of 160 residents of a retirement home in Southern Germany died of COVID-19 and 37 death patients are attributed to an undetected hospitalized case in Eastern Germany [18, 19]. Furthermore, Zhan et al. report that HCW represent 4.4% of all patients with COVID-19 in China [12]. Additionally, they outline that 23 HCW died due to COVID-19 with only 2 of 23 HCW been specifically assigned to treat COVID-19 patients, strengthening the high risk associated with inadequate hygienic measures and insufficient protection during early stages of the pandemic [12]. Especially, surgical disciplines bear the high risk of close contact exposures for HCW during intubation, surgical treatment and dressing change. While our patient may have benefited from the hospital’s measures to prevent COVID-19, we believe that particularly the routinely performed contact precautions due to pending MDRO screening results helped to prevent an intra-hospital transmission of SARS-CoV-2. If MDRO test results had been available upon admission, the patient would have been placed in a multi bedroom, allowed to move freely on the ward and treating HCW would not have been obliged to wear gloves and protective gowns when entering the patient’s room. When MDRO test results arrived 4 days after admission, the patient’s respiratory condition had already worsened so that he was placed in a single bedroom on our IMC (Fig. 3, Room 2). On our IMC, HCW are specifically assigned to a patient, which may have further helped to prevent a nosocomial spread. At least in our case, these simple and well-established contact precautions seemed to be sufficient to prevent nosocomial spread of SARS-CoV-2—even in high-risk exposed HCW.

A similar scenario of an undetected case of COVID-19 was also described by Wong et al. who report the case of a 64-year-old female patient who remained undetected for 35 h and had contact to 72 HCW as well as 49 patients [20]. When comparing both scenarios, it is especially remarkable how many contacts and exposures can be set within a short period within a hospital: 72 HCW and 49 patients in just 35 h (Wong et al.) and 66 HCW in 6 days in our patient who was already under protective isolation. Contrary to our study, only HCW and patients with fever and/or respiratory symptoms were tested in the study of Wong et al. which resulted in 52 oropharyngeal swabs of which all turned out to be negative [20].

## Conclusion

Hygienic measures and contact precautions aimed at preventing the spread of MDRO may have helped to prevent a SARS-CoV-2 transmission to HCW—despite high-risk exposure during intubation, surgical treatment and dressing change.

## Abbreviations

COPD: Chronic obstructive pulmonary disease
COVID-19: Coronavirus-induced disease
HCW: Healthcare workers
IMC: Intermediate care unit
SARS-CoV-2: Severe acute respiratory syndrome coronavirus 2
MDRO: Multi-drug-resistant organisms
MRSA: Methicillin-resistant *Staphylococcus aureus*
PHILOS: Proximal humerus internal locking system
*R*_0_: Reproduction number
RdRp: RNA-dependent RNA polymerase
RKI: Robert Koch Institute
VRE: Vancomycin-resistant enterococci
WHO: World Health Organization
